# The *GTPase Activating Rap/RanGAP Domain-Like 1* Gene Is Associated with Chicken Reproductive Traits

**DOI:** 10.1371/journal.pone.0033851

**Published:** 2012-04-09

**Authors:** Xu Shen, Hua Zeng, Liang Xie, Jun He, Jian Li, Xiujuan Xie, Chenglong Luo, Haiping Xu, Min Zhou, Qinghua Nie, Xiquan Zhang

**Affiliations:** 1 Department of Animal Genetics, Breeding and Reproduction, College of Animal Science, South China Agricultural University, Guangzhou, Guangdong, China; 2 Guangdong Provincial Key Lab of Agro-Animal Genomics and Molecular Breeding, Guangzhou, China; 3 Institute of Animal Science and Veterinary, Hainan Academy of Agricultural Sciences, Haikou, Hainan, China; 4 Institute of Animal Science, Guangdong Academy of Agricultural Sciences, Guangzhou, Guangdong, China; 5 Biotechnology Institute, Jiang Xi Education College, Nanchang, Jiangxi, China; VIB & Katholieke Universiteit Leuven, Belgium

## Abstract

**Background:**

Abundant evidence indicates that chicken reproduction is strictly regulated by the hypothalamic-pituitary-gonad (HPG) axis, and the genes included in the HPG axis have been studied extensively. However, the question remains as to whether any other genes outside of the HPG system are involved in regulating chicken reproduction. The present study was aimed to identify, on a genome-wide level, novel genes associated with chicken reproductive traits.

**Methodology/Principal Finding:**

Suppressive subtractive hybridization (SSH), genome-wide association study (GWAS), and gene-centric GWAS were used to identify novel genes underlying chicken reproduction. Single marker-trait association analysis with a large population and allelic frequency spectrum analysis were used to confirm the effects of candidate genes. Using two full-sib Ningdu Sanhuang (NDH) chickens, *GARNL1* was identified as a candidate gene involved in chicken broodiness by SSH analysis. Its expression levels in the hypothalamus and pituitary were significantly higher in brooding chickens than in non-brooding chickens. GWAS analysis with a NDH two tail sample showed that 2802 SNPs were significantly associated with egg number at 300 d of age (EN300). Among the 2802 SNPs, 2 SNPs composed a block overlapping the *GARNL1* gene. The gene-centric GWAS analysis with another two tail sample of NDH showed that *GARNL1* was strongly associated with EN300 and age at first egg (AFE). Single marker-trait association analysis in 1301 female NDH chickens confirmed that variation in this gene was related to EN300 and AFE. The allelic frequency spectrum of the SNP rs15700989 among 5 different populations supported the above associations. Western blotting, RT-PCR, and qPCR were used to analyze alternative splicing of the *GARNL1* gene. RT-PCR detected 5 transcripts and revealed that the transcript, which has a 141 bp insertion, was expressed in a tissue-specific manner.

**Conclusions/Significance:**

Our findings demonstrate that the *GARNL1* gene contributes to chicken reproductive traits.

## Introduction

Egg number at 300 d of age (EN300), age at the first egg (AFE), and brooding behavior are valuable indices of chicken reproductive ability. In female chickens, sexual maturity is usually expressed as AFE. The AFE trait has been under artificial selection to enhance egg production efficiency [1]. EN300 is another reproductive trait of economic importance, while incubation behavior also affects egg production, as it results in the cessation of egg laying [2]. Chicken reproduction is controlled by photoperiod [3]. Generally, the process of chicken egg production is strictly regulated by the hypothalamic-pituitary-gonad (HPG) axis [4]. Gonadotrophin releasing hormone (GnRH) and its receptor (GnRHR) start the cascade, and neuropeptide Y (NPY) is known to inhibit GnRH secretion via its receptor (Y1R) and to control ovulation [5]. Under photo-stimulation, GnRH is synthesized, secreted by the hypothalamus and binds to its receptor, which stimulates the pituitary gland to secret gonadotrophins that evoke steroid synthesis in the gonad, regulating ovarian follicle growth and ovulation in hens [6], [7]. The hypothalamic vasoactive intestinal peptide (VIP) - pituitary prolactin (PRL) neuroendocrine pathway also controls reproductive cycles via dopaminergic neurotransmission in avian HPG system [8]–[10]. PRL is a key hormone that is absolutely necessary for egg laying and incubation behavior in poultry [11], [12]. After stimulation by VIP, PRL inhibits the release of gonadotropins and thereby induces and maintains chicken incubation behavior [13]–[15].

The genetic mechanism behind incubation behavior has been widely studied because of its potential effect on egg production. This mechanism is a polygenic trait that is controlled by a set of autosomal genes [16]. Genes in the HPG axis showed high association with reproductive traits such as broodiness and egg production [9], [17]–[30], however, this association depends on the population used [22], [30]. Aside from the genes distributed in HPG axis, other novel genes have been discovered to affect chicken reproduction traits [31]–[33].

Several approaches have been applied to identify the novel genes involved in chicken reproduction. A genome-wide scan is a powerful approach to understanding this complex trait. Quantitative trait loci (QTLs) for egg number, egg production rate, AFE and broodiness were identified through genome-wide scans [34]–[42]. Genome-wide association studies based on high density SNPs can be performed to detect QTLs that could not be detected by previous studies based on microsatellite genotyping [43]–[47]. A genome-wide association study attempts to obtain information on all variants, but a gene-centric SNP approach would be efficient enough to capture SNPs associated with particular traits [48], [49].

Transcriptome profiling can be also used to identify new genes associated with chicken reproductive traits. Although many studies on the genetic effects of candidate chicken reproduction genes have been reported, few studies have reported transcriptomic and proteomic changes. In previous studies, transcripts related to high-egg production were identified by suppressive subtractive hybridization analysis (SSH), and several of the identified transcripts were further confirmed to be significantly increased in hens with higher egg production, though they were not part of the HPG axis [50]–[52]. Therefore, it is valuable to identify novel genes related to chicken reproduction.

The aim of the present study is to identify novel genes involved in chicken reproductive traits using SSH analysis, an Illumina 60K chicken Beadchip GWAS, and a gene-centric GWAS, with confirmation via analysis of single marker-traits, allelic frequency spectra, and alternative splicing.

## Results

### *GARNL1* identified as a candidate gene underlying chicken broodiness by suppression subtractive hybridization

**Figure 1:**
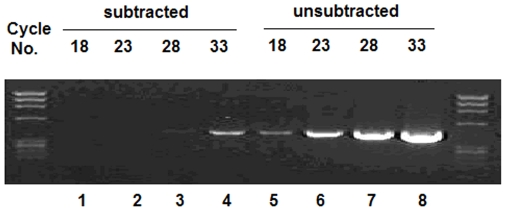
Determination of subtraction efficiency. The chicken housekeeping gene G3PDH was amplified from the subtracted sample (showed in lane 1–4) and the unsubtracted sample (showed in lane 5–8). The number above the lane represents the PCR cycle.

**Figure 2:**
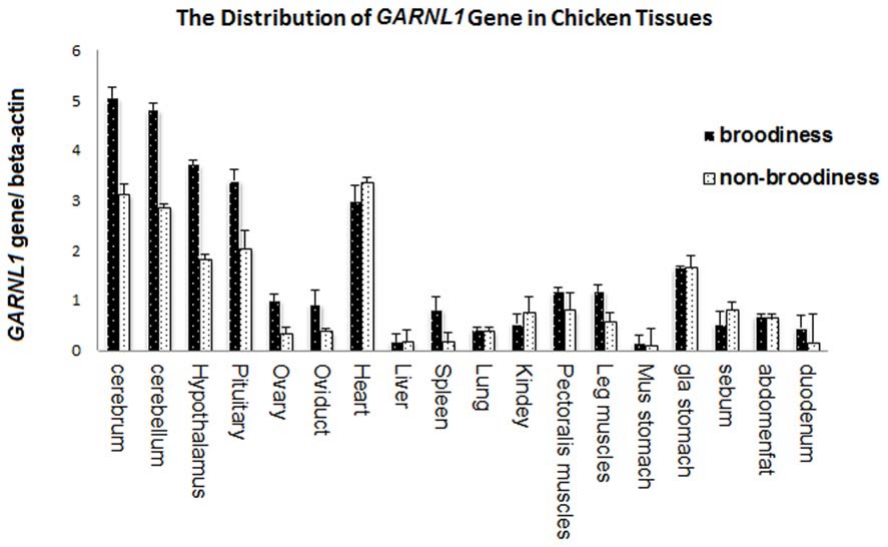
Comparison of GARNL1 mRNA expression level between brooding and non-brooding chickens. qPCR was performed to validate the mRNA level of the GARNL1 gene between the brooding and egg-laying stages in NDH chickens. The horizontal axis indicates the tissues used for detection, and the vertical axis indicates the 2^−ΔCt^ value (shown as average ± SEM).

A subtraction library was made by subtracting cDNA from the pituitary at the egg-laying stage. As shown in Figure 1, construction of the pituitary-subtracted cDNA libraries was successful. Genes differentially expressed between brooding and non-brooding chicken pituitary glands were enriched for and sequenced, and 57 annotation transcripts and 20 unknown transcripts were characterized (Table S1). Gene ontology (GO) analysis was performed to investigate the functions of the putatively differentially expressed transcripts. Biological process accounted for the major portion of GO annotations, compared with cellular component and molecular function. Among the category of biological process, genes were involved in processes such as eye photoreceptor cell development, ovarian follicle development, epinephrine biosynthesis, regulation of small GTPase mediated signal transduction, G-protein coupled receptor protein signaling pathways, and so forth (Table S2). On the basis of biological process annotations, 10 transcripts were selected to be validated by qPCR. Among the 10 transcripts, one was identified as belonging to the chicken *GARNL1* gene (Figure S1). The *GARNL1* gene was differentially expressed between tissues (Figure 2). Low levels of mRNA expression were detected in the ovary, oviduct, liver, spleen, lung, kidney, muscular stomach, sebum, abdomen fat, and duodenum; however, higher expression levels were observed in the cerebrum, cerebellum, hypothalamus, pituitary, heart, and glandular stomach. Gene expression levels in the cerebrum, cerebellum, hypothalamus, pituitary, ovary, oviduct and spleen were significantly higher in broody chickens than in non-broody chickens (P<0.05), with the levels in tissues from broody chickens 1.6 times to 4.3 times higher than those of non-broody chickens. In contrast, *GARNL1* expression in leg muscle was 2-fold higher in non-broody chickens.

### GWAS indicates that SNPs associated with chicken reproductive traits are located in the *GARNL1* gene

Before GWAS analysis was carried out, stratification analysis was conducted in the two-tail sample. The IBS was not significantly different between two tails sample (P_permu_<0.05). In all, 2802 SNPs were associated with EN300 in the NDH two tail sample at the 5% genome-wide level (validated by 10000 permutation tests), and of this total, 470 SNPs were at significant at the 1% level (Table S3). On chicken chromosome 5, 118 SNPs were associated with EN300 (Table S3). Among the 118 EN300-associated SNPs, rs14533299 and GgaluGA282818 composed a haplotype block. The linkage distance in this block is 1691 kb, and the *GARNL1* gene was observed to be located within this block (Figure S2).

### Gene-centric GWAS reveals an association of several SNPs in the *GARNL1* gene with chicken EN300 and AFE

Six SNPs were highly significantly associated with both EN300 and AFE (P_permu_<0.05 and P_permu_<0.01) (Table 1). A SNP cluster located on chromosome 5 was associated with both AFE and EN300 in another NDH two tail sample. Among the SNP cluster, 5 SNPs were located in the *GARNL1* gene.

**Table 1:** The 6 SNPs significantly associated with both EN300 and AFE in a two-tail sample.

Chr^1^	SNP ID	Gene	Chr: bp	Type	EN300	AFE
					P_permu_ Value	P_permu_ Value
5	rs16492011	GARNL1	38621623	intron_39	0.02699^*^	0.01099^*^
5	rs15701085	GARNL1	38674045	exon_26	0.02849^*^	0.004998^**^
5	rs16492034	GARNL1	38680830	intron_20	0.008496^**^	0.04598^*^
5	rs13585983	GARNL1	38681662	exon_20	0.009495^**^	0.03998^*^
5	rs14532787	GARNL1	38699909	exon_15	0.002999^**^	0.02899^*^
24	rs16199186	NCAM1	5972092	exon_5	0.0009995^**^	0.02399^*^

1 The chromosome where the associated SNPs located.

* and ** indicate Ppermu<0.05, and Ppermu<0.01, respectively.

The association of *GARNL1* SNPs with chicken EN300 and AFE was further analyzed in a NDH population comprising 1301 individuals. As showed in Table 2, corrected by SLIDE, rs15700989 was significantly associated with EN300 (P<0.01) and rs15701085 was associated with AFE (P<0.05).The block composed of rs14532787 and rs14532779 was also significantly associated with EN300 (P = 0.0088) (Table 3). In this block, there are 4 haplotypes, including H1 (TG, 70.1%), H2 (TC, 5.5%), H3 (CG, 23.3%), and H4 (CC, 1.1%). H2H2 and H2H4 had higher EN300 than the other diplotypes.

**Table 2:** Association of 17 SNPs with chicken reproductive traits in population.

SNP	Information			EN300 trait		AFE trait	
	Position^1^	Location^2^	Allele	Pointwise-P	Corrected-P	Pointwise-P	Corrected-P
rs15700949	38617982	3′flanking	A/G	0.5737	1.0000	0.2336	0.9854
rs16492011	38621623	intron 39	G/C	0.3796	0.9994	0.1031	0.8171
rs14532750	38624284	intron39	T/C	0.0191	0.2667	0.4148	0.9998
rs15700989	38648067	intron 37	A/G	0.0001	0.0023^**^	0.09498	0.7912
rs16492027	38654577	intron 36	T/C	0.3907	0.9996	0.05072	0.5644
rs16492031	38660349	intron 32	A/G	0.6481	1.0000	0.2189	0.9793
rs15701085	38674045	exon 26	G/A	0.0621	0.6365	0.002894	0.0496^*^
rs13585983	38681662	exon 20	A/G	0.5698	1.0000	0.1158	0.8505
rs16492034	38680830	intron 20	T/C	0.1092	0.8347	0.03888	0.4716
rs14532779	38697653	intron 15	T/C	0.0876	0.7614	0.1711	0.9461
rs14532787	38699909	exon 15	T/C	0.0316	0.4002	0.02979	0.3819
rs16492056	38708121	intron 8	T/C	0.0190	0.2656	0.1233	0.8695
rs15701119	38711935	intron7	TTAAA/-	0.4808	0.9999	0.3975	0.9996
rs14532824	38730001	5′flanking	T/C	0.4837	0.9999	0.7553	1
rs14532808	38724344	intron1	A/G	0.9870	1.0000	0.3928	0.9996
rs14532819	38726759	intron1	T/A	0.2698	0.9928	0.6031	1
rs14532831	38731847	5′flanking	T/C	0.1901	0.9626	0.03088	0.3935

1 The position of the site on chromosome 5 in coordinates from the chicken genome database at UCSC (http://genome.ucsc.edu/cgi-bin/hgBlat?command=start).

2 the location of the variants found inside the *GARNL1* gene.

Pointwise P indicated the P value gained by PLINK and Corrected-P means the P value corrected by SLIDE, ^*^ and ^**^ indicate P<0.05, and P<0.01, respectively.

**Table 3:** The association of haplotypes composed of rs14532787 and rs14532779 with EN300 traits.

Trait	P value	H1H11(647)	H1H21(102)	H1H31(408)	H2H21(5)	H2H31(53)	H2H41(2)	H3H31(76)	H3H41(1)
EN300	0.0086**	92.79±1.09^bB^	93.07±2.64^b^	94.06±1.32^bB^	132.31±11.63^aA^	96.84±3.72^a^	131.73±18.44^a^	98.49±3.05^Ab^	106.06±25.87^a^

Values were expressed as least-square means ± standard errors (SE).

The number in brackets was the number of chickens tested for each diplotype.

** indicate P<0.01.

The ^a,^ ^b^ or ^A,^ ^B^ values with no common superscripts within a column for each site that differed significantly (P<0.05) or highly significantly (P<0.01).

### Allelic frequency spectrum of the chicken *GARNL1* gene

Allelic frequencies of rs15700989 were different among the 5 populations. The frequency of rs15700989 was 1.0 in Leghorn layers (Table 4), with a highly significant difference between Leghorn layer and the other 4 native Chinese chickens. The chi-square test values for the genotype distribution of rs15700989 showed significant difference between Leghorn layer and the other 4 Chinese native chickens (P<0.01) (Table 5), in accordance with their egg-production performance.

**Table 4:** Allelic frequencies of rs15700989 in the GARNL1 gene in the 5 chicken populations.

Site	Allele	LH(n = 60)	BEH(n = 41)	NDH(n = 82)	XH(n = 50)	RJF(n = 33)
rs15700989	G	1	0.352	0.31	0.09	0.22

LH = Leghorn layers, BEH = Baier Huang chickens, NDH = Ningdu Huang chicken, XH = Xinghua chicken, RJF = Red Jungle Fowl.

The number in brackets was the number of chickens used.

Hardy-Weinberg equilibrium was set at the 0.01 level.

**Table 5:** Chi-square test of genotype frequency for rs15700989 in the 5 populations.

Site	Populations	χ^2^ Value^1^			
		RJF	XH	LH	NDH
rs15700989	BEH	0.14	27.35**	101.00**	5.27
	RJF		21.22**	93.00**	3.29
	XH			119.00**	20.64**
	LH				120.79**

1 χ^2^_0.05_(df = 1) = 3.841; χ^2^_0.01_(df = 1) = 6.635; χ^2^_0.05_(df = 2) = 5.991; χ^2^_0.01_(df = 2) = 9.21;

* P<0.05;

** P<0.01.

### Alternative splicing of the chicken *GARNL1* gene

**Figure 3:**
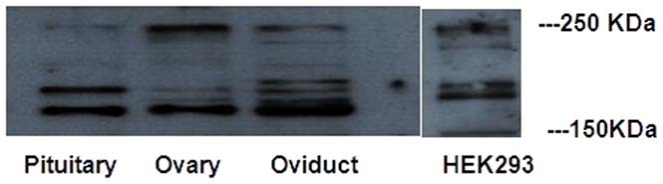
Western blot analysis of GARNL1. The Western blot analysis revealed 4 isoforms of chicken GARNL1 within the chicken pituitary, 6 isoforms within the chicken ovary, and 7 isoforms within the chicken oviduct. Their molecular weights ranged from 150 KDa to 250 KDa. In human HEK293, 6 isoforms were detected.

The chicken *GARNL1* gene is predicted to be located on chromosome 5 and to span positions 38,617,769–38,729,036 on the reverse strand, with a total gene size of 111,268 bp. Four, six, and seven isoforms from the pituitary, ovary, and oviduct, respectively, could be detected by Western blotting. The molecular weights of these isoforms ranged from 150 KDa to 250 KDa (Figure 3).

Five alternatively spliced transcripts, *GARNL1-w* (NCBI accession number: JF330255), *GARNL1-v1* (NCBI accession number: JF330256), *GARNL1-v2* (NCBI accession number: JF330257), *GARNL1-v3* (NCBI accession number: JF330258, and *GARNL1-v4* (NCBI accession number: JF330259) were detected in the cDNA pool prepared from cerebrum, cerebellum, hypothalamus, pituitary, ovary, and oviduct tissues. Five transcripts were generated as a result of exon skipping and intron inclusion (Table 6 and Figure S3). The wild-type transcript, *GARNL1-w*, which is composed of 41 exons and 40 introns, was successfully cloned. The complete coding sequence of *GARNL1-w* is 6,108 bp long and encodes 2,035 amino acids. Chicken GARNL1 shares a high amino acid sequence identity with those of human (89.3% with AY596971, 89.4% with AY596970), mouse (87.4% with AY596972, 87.6% with AY596972), and zebrafish (73.2% with AB476643, 74.3% with AB476644), and it is predicted to be a nuclear protein (with 63% probability). Similar to the human *GARNL1* gene and the mouse *GARNL1* gene, all 5 transcripts contain a Rap/Ran-GAP domain (AA 1825–AA 2004), two transmembrane helices (AA 1203–AA 1225, AA 1385–AA 1407), and a leucine zipper motif (AA 1068–AA 1089), but have lost the N-terminal coiled coil domain (shown in figure S4).

**Table 6:** The alternative splicing types of the chicken *GARNL1* gene.

SequenceID	Length^1^	ExonNumber	IntronNumber	Amino Acid^2^	MolecularWeight	Types of AS^3^
*GARNL1-w*	6108	41	40	2035	230 KD	/
*GARNL1-v1*	6261	40	39	2086	235 KD	Exon Skipping (exon 40, 31 bp)
*GARNL1-v2*	6405	41	40	2135	240 KD	Intron inclusion (fragment of intron 16, 141 bp) andExon Skipping (exon 40, 31 bp)
*GARNL1-v3*	5995	40	39	1984	225 KD	Exon Skipping (exon 21, 153 bp)
*GARNL1-v4*	6609	42	41	2203	247 KD	Intron inclusion (fragment of intron 16, 141 bp and fragment of intron 19, 201 bp)

1 Length of the open reading frame (ORF).

2 The number of the amino acid coded by the chicken GARNL1 gene.

3 The type of alternative splicing observed.

**Figure 4:**
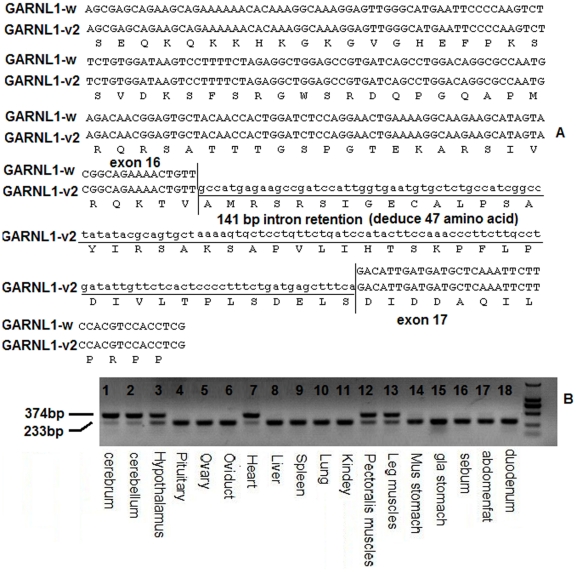
Tissue-specific analyses of GARNL1 transcripts. (A). Partial cDNA and deduced amino acid sequence of the 141 bp insertion. Lower-case letter indicated the 141 bp insertion sequence from intron 16. (B). The distribution of transcripts containing the 141 bp insertion, which was only expressed in six tissues; the cerebrum, cerebellum, hypothalamus, heart, pectoral muscle, and leg muscle.

**Figure 5:**
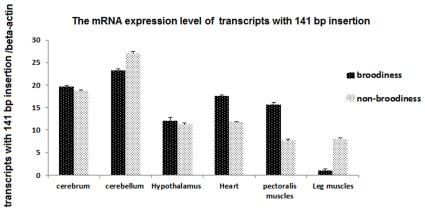
Expression level of transcripts with the 141 bp insertion. The horizontal axis and vertical axis indicate different tissues and 2^−ΔCt^ value (mean ± SEM), respectively.

The variant *GANRL1-v2* (deduced to encode a 2134 AA peptide) skips exon 40 and includes a 141 bp intron sequence between the exon 16 and exon 17. RT-PCR showed that the 141 bp intron inclusion was tissue specific, being observed only in the cerebrum, cerebellum, hypothalamus, heart, pectoral muscle, and leg muscle. Its mRNA expression level was higher than the other isoforms without 141 bp intron inclusion (Figure 4). Similarly, the *GARNL1-v4* transcript contained a 201 bp fragment of intron 19, and a single amino acid change, N (Asn) to D (Asp), occurs at the new exon-exon junction. *GARNL1-v4* mRNA with the 201 bp intron fragment was present at very low levels (data not shown). The mRNA expression levels of transcripts with the 141 bp intron inclusion sequence (Figure 5) in the cerebrum, cerebellum, and hypothalamus were almost the same between brooding and non-brooding chickens. However, its expression levels in heart and pectoral muscles of the brooding chickens were 1.5 and 2 times greater than those of the non-brooding chickens, respectively. In leg muscle, the expression was 8-fold higher in the non-brooding chickens than in brooding chickens.

## Discussion

In this study, data from a SSH analyses, a GWAS, and a gene-centric GWAS indicate that the *GARNL1* gene is involved in reproduction and that some *GARNL1* variants are associated with chicken reproductive traits.

The SSH analysis indicated that the *GARNL1* gene was involved in chicken brooding behavior. Comparing to the digital gene expression methods, such as RNA-seq, SSH is not a prevailing experimental method for detecting differentially expressed genes. SSH have several limitations, relatively low throughput, highly false positives, and generally not statistical significance. But the following qPCR validation would help to get some good results with the following experiment validation [53]–[55]. This result was consistent with previous findings. Chicken *GARNL1* was identified as being potentially related to high egg production in Taiwan Country chickens [50], and higher *GARNL1* expression levels have been observed in high egg producing strains [51]. Furthermore, the mRNA level of the *GARNL1* gene was specifically associated with total egg number at 500 d of age or egg rate after the first egg [52]. The cerebellum was found to have the highest expression level of human *GARNL1* gene among the brain tissues, corresponding to its influence on 14q13-linked neurological phenotypes [56]. In zebrafish, *GARNL1* was a strong candidate gene for brain developmental delay [57]. In our study, the expression of chicken *GARNL1* gene varied at different stages. We found *GARNL1* to be predominantly expressed in the brain, and the levels of the *GARNL1* gene were consistently higher in the hypothalamus, pituitary, ovary, and oviduct of broody hens. The expression level of transcripts that included the 141 bp intron sequence suggested that the cerebellum may be an important action region in chickens and the variants of *GARNL1* do not impair their function on chicken reproductive traits. In conclusion, the expression levels of the *GARNL1* gene could reflect its functions in chicken reproduction.

Two tail samples were used to detect SNPs associated with broodiness and EN300 in this study. The first QTLs for broodiness were recently detected in a region within 95 cM of GGA5 [42], where the *GARNL1* gene is located. In the present study, the haplotype block between rs14533299 and GgaluGA282818 was also shown to be related to EN300 (data not showed). The *GARNL1* gene is located in this region. Among all 25 protein-coding genes located on this region, the *GARNL1* gene was the only one that has been reported to be related to reproductive traits in mRNA level in chickens [50]–[52]. Therefore, the *GARNL1* gene may be associated with reproduction in chickens.

As the *GARNL1* gene might be involved in chicken reproduction, its polymorphisms could be related to chicken reproductive traits. However, no studies on the association between the mutations of *GARNL1* gene and chicken reproductive traits were carried out. In humans, the *GARNL1* gene was an important candidate gene for human 14q13 deletion phenotypes, and two mutations in *GARNL1* were identified in a family with idiopathic basal ganglia calcification [56]. Polymorphisms of the *GARNL1* gene were associated with both EN300 and AFE in a two tail sample in our gene-centric association analysis. This result confirmed our previous SSH findings and was validated in a large population. An analysis of the allelic frequency spectra of *GARNL1* SNPs further supported the association. The frequencies of the rs15700989 were associated with EN300 associated with divergent egg production performance, and the frequency of predominant alleles of rs15700989 was 1.0 in Leghorn layer and was descending in Leghorn, BEH, NDH, XH, and RJF. The predominant allele of rs15700989 was related to higher EN300 trait in NDH population. Thus, the allelic frequency data supports the conclusion that the chicken *GARNL1* gene contributes to chicken reproduction. The block composed of rs14532787 and rs14532779 was significantly associated with EN300 traits. Although both of them were not showed significantly relationship with EN300 after corrected by SLIDE in single marker association, the CC genotype of rs14532787 resulted in a higher EN300 and an earlier AFE than did the other two genotypes (Table S4). However, the genotype CC can be only observed in the NDH population. Compared to the variance of total egg number at 40 week between early sexual mature group and later sexual mature group in Leghorn layer [58], rs14532787 might undergo artificial selection in NDH population, aiming at the increase of egg production by early sexual mature and shortening the interval of oviposition. These two SNP may contribute to EN300 by interacting each other.

Further analysis of the organization, tissue expression, and alternative splicing of the chicken *GARNL1* gene was conducted. Using Western blotting, 5 alternatively spliced transcripts of the *GARNL1* gene were isolated from chickens in this study. Note that none of the alternative splicing isoforms had impaired protein domains. Chicken GARNL1 is conserved with mammals, but it has some unique features. A variant of the human GARNL1 lacking exon 40, has been found and corresponded to GARNL1-v1 in chicken [56]. Chicken GARNL1 has lost the N-terminal coiled coil domain and subsequently the ability to bind to other proteins. In mice, GARNL1 plays a crucial role during brain formation and maintenance. A partial murine GARNL1 product identified as GRIPE (GAP-related interacting protein to E12) binds to the helix-loop-helix domain of transcription factor E12 and regulates E12-dependent target gene transcription [59]. Similar to the murine GRIPE, the region responsible for binding to HLH domains was present in all isoforms of chicken GARNL1. The Rap/Ran-GAP domain is widely distributed in signaling proteins [60]–[62], and two arginine residues in Rap/Ran-GAP domain are important for the GAP activity of GRIPE in mice [59]. Two arginine residues were found in the Rap/Ran-GAP domain of chicken GARNL1.

In conclusion, we reveal that the chicken *GARNL1* gene has an important effect on chicken reproductive traits, as determined from the data from SSH analyses, GWAS, and gene-centric GWAS. This effect was validated by analysis of allele frequency spectra, and further characterization of several aspects of the gene and its expression.

## Materials and Methods

### Ethics Statement

The study was approved by the Animal Care Committee of South China Agricultural University (Guangzhou, People's Republic of China) with approval number SCAU#0011. Animals involved in this study were humanely sacrificed as necessary to ameliorate their suffering.

### Identifying candidate genes underlying chicken broodiness by SSH analysis

A pair of full-sib female NDH chickens was used for the suppression subtractive hybridization (SSH) experiment. One individual was broody, and its brooding lasted for more than 7 d, and the other one was continuously laying. The pituitary gland and 17 other tissues were collected after the brooding individual had been incubating for 10 d. At that time, the chicken's incubation behavior was quite typical, and both ovary and oviducts were atrophied. The laying chickens' tissues were also collected at the same time.

Total RNA was extracted from the tissues using Trizol reagent (Invitrogen, California, USA) according to the manufacturer's protocol. Total RNA was treated with RNase-free DNaseI (Takara, Osaka, Japan) for 45 min at 37°C to ensure that it was free of DNA contamination. RNA quantity and quality were assessed using a Thermo Scientific Nanodrop1000 spectrophotometer (Nanodrop Technologies, Wilmington, Delaware, USA) and by formaldehyde denaturation agarose gel electrophoresis.

Suppression subtractive hybridization was performed with an equal amount of tester mRNA (2 µg) from the brooding stage, as well as the driver mRNA from the egg-laying stage. Following the manufacturer's protocol for the PCR-Select cDNA Subtraction Kit (Clontech, Palo Alto, CA, USA), after two subtraction hybridizations and two suppression PCRs, the subtraction efficiency was evaluated by PCR using primers for the chicken house-keeping gene G3PDH (P#1, Table S5). cDNAs were cloned and inserted into the pMD20-T vector (Takara, Osaka, Japan) and were then transferred into chemically competent *E. coli* (JM109) cells to generate SSH libraries.

Subtractive products longer than 300 base pairs were picked for sequencing by Invitrigen Co. Ltd (Shanghai, China). The vector nucleotide sequences were removed, and the remaining sequences clustered into contigs using DNAstar software. The basic local alignment search tool (BLAST) http://blast.ncbi.nlm.nih.gov/ was used for identifying and annotating genes.

Quantitative real-time PCR (qPCR) was performed with the Agilent Stratagene Mx QPCR Instrumentation (Agilent Technologies, Wilmington, DE, USA) for follow-up of candidate genes, using the SsoFast EvaGreen Supermix (Bio Rad Laboratories, Hercules, CA, USA). The four individual cDNAs were used as templates for qPCR amplification. The primers used for the qPCR were designed using Primer Express 2.0 software (Applied Biosystems, Foster City, CA, USA). A housekeeping gene, the chicken beta-actin gene (accession: L08165), was used as internal control. Therefore, two sets of primers (P#2 and P#3, Table S5) were designed and used for the qPCR amplification. Each reaction mixture contained 10 µL of Eva Green PCR Master Mix, 1 µL of each primer (10 µM), 7 µL of RNase-free water and 1 µL of cDNA in a final volume of 20 µL. Standard amplification conditions were as follows: 95°C for 30 s, 40 cycles of 95°C for 5 s, 60°C for 30 s. Fluorescent signal was collected after an extension at 65°C in each cycle. Chicken *GARNL1* gene relative expression was calculated by 2^−ΔCt^ method, and ΔCt = Ct_target gene_−Ct_β-actin_.

### GWAS for a two tail sample using 60K chips

The age of the first egg (AFE) and the total number of eggs at 300 d of age (EN300) were recorded in a breeding population kept in Guangdong Wens' Foodstuff Co. Ltd (Guangdong, China). Twenty Ningdu Sanhuang (NDH) female chickens from the above population were divided into 2 groups. Group 1 was composed of the 10 individuals with the highest EN300 values (an average of 145 eggs) and no observable incubation behavior, and group 2 was composed of the 10 individuals that had the lowest EN300 values (an average of 66 eggs) as well as an average duration of broodiness of 51 d. Twenty Illumina 60K chicken chips were used for the two-tailed association study.

Stratification analysis was performed to detect the IBS of the two-tail sample before GWAS studies were carried out. SNP quality control metrics were analyzed using GenomeStudio software (version 2009.1). A SNP was removed if its call rate was less than 100%, or its minor allele frequency (MAF) was less than 5%, and its Hardy-Weinberg equilibrium (HWE) p-value was too low (P<0.00001). As a result, 54,424 SNPs were selected for use in the GWAS. PLINK was used for the single-marker association analysis. PLINK single marker basic allelic association (X_1_^2^) tests (the –assoc option) were performed for each of the post-QC SNPs. PLINK's max (T) permutation procedure (the –mperm option) was set to 10000 for the two-tailed test, in order to get accurate P values by reducing false positives. The SNPs detected by PLINK were used to analyze haplotype structure with Haploview 4.1 software http://www.broad.mit.edu/mpg/haploview/.

### Gene-centric GWAS

Ninety-six NDH chickens were selected on the basis of AFE and EN300 measurements and genotyped for 384 SNPs using the Illumina GoldenGate™ iSelect Array genotyping platform (BGI, Shenzhen, China) via a commercial service. The 384 SNPs were located in an AFE QTL region or in 20 novel candidate genes selected according to previous reports [18], [22]–[26], [35], [36], [38], [51], [52], [63] (Table S6). The DNA sample set included 3 replication pairs. Twenty-four early sexual mature individuals with an AFE of 91 to 95 d and 24 late sexual mature individuals with an AFE of 160 to 179 d, and 24 low production individuals with an EN300 of 1 to 22 eggs and 24 high production individuals with an EN300 of 140 to 163 eggs were used in the gene-centric genome-wide association study. The average AFE value in the early group was 92.5 d, whereas in the late group it was 166.4 d. The average EN300 value in the low group was 28 eggs, and in the high group, 146 eggs. The gene-centric GWAS association analysis was performed by quantitative trait association analysis using PLINK with 10000 permutations.

### Marker-trait association analysis in NDH female population

A total of 5 site-specific primers were designed by Assay design and synthesis by the Sangon Biotech Company (Shanghai, China), a commercial service. We validated the SNPs for which associations were found using the Sequenom genotyping platform. The effects of an additional 12 SNPs on EN300 or AFE were investigated by an association study using the PCR-RFLP method in the NDH population (P#5 to P#16, Table S5). PCR was performed in a 10 µL reaction mixture containing 1 µL of Taq polymerase (Dongsheng Co., Guangzhou, China), 5 µL of the 2×PCR buffer supplied by the manufacturer, 1 µM of each primer, 50 ng genomic DNA, with ddH_2_O added to a total volume of 10 µL. The PCR program used was 3 min at 94°C, followed by 32 cycles of 30 s at 94°C, 30 s at 59°C, 45 s at 72°C, and a final extension of 5 min at 72°C in a Bio-Rad Mycycles (Bio-Rad Laboratories, Hercules, CA, USA). PCR products were digested in a 37°C or 65°C water bath overnight with *MspI*, *HindIII* (2 sites), *DraI* (2 sites), *SacII*, *KpnI*, *StuI*, *PvuII*, *TaqI*, *Csp6I*, and *BsuRI*. Genotypes were determined by electrophoresis after restriction digestion.

PLINK (version 1.07) was used to perform analysis for evaluating genetic effect of 17 sites of *GARNL1* gene on EN300 and AFE. Sliding-window method for Locally Inter-correlated markers with asymptotic Distribution Errors corrected (SLIDE) program [64] was used to correct the P value, and all the point-wise P value were corrected based on 10000 sampling. A P value ≤0.05 was considered significant in the analyses. Multiple comparisons were conducted with least squares means using Fisher's least significant difference method.

The Hardy-Weinberg equilibrium and haplotype structure were analyzed using Haploview 4.1 software [65]. Haplotypes were constructed on the basis of genotype data using PHASE 2.0 software (http://en.wikipedia.org/wiki/Phase).The minimum haplotype frequency was set to 1%. Association analyses of haplotypes with the EN300 and AFE were carried out using SAS GLM procedure (SAS Institute Inc., Cary, NC, USA) with the following model
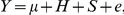
Where *Y* is a trait observation, *μ* is the overall population mean, *H* is the effect of haplotype, *S* is the fixed effect of sire, and *e* is the residual error.

### Allelic frequency spectrum of the chicken *GARNL1* gene

Five populations, Red Jungle fowl (RJF) (n = 33), Baier Huang chicken (BEH) (n = 41), White Leghorn (LH) (n = 60), Xinghua chicken (XH) (n = 50), and Ningdu Sanhuang chicken (NDH) (n = 82), were genotyped at the reproduction-associated SNPs of the *GARNL1* gene to obtain allele frequency spectra. RJF, XH and NDH showed low egg-production ability, with 60∼130 eggs per year because of their intractable incubation behavior (100% incidence of broodiness in RJF, 70∼80% incidence of broodiness in XH and 50∼60% incidence of broodiness in NDH). BEH chickens had a 10∼15% incidence of broodiness and an egg-production of 180 per year. The Leghorn chicken is a famous layer breed with excellent egg-production ability and no incubation behavior. The primers used were the same as those used for genotyping the NDH population.

Chi-square (χ^2^) tests performed on a 2×3 (or n) contingency table. A P≤0.05 was considered significant in all analyses.

### Analysis of *GARNL1* alternative splicing isoforms

Total protein was collected using Trizol reagent (Invitrogen, California, USA) according to the manufacturer's protocol. Proteins were separated on an 8% SDS-PAGE gel by electrophoresis and transferred to PVDF membranes (Millipore, Billerica, MA, USA). Membranes were blocked using TBST containing 5% nonfat milk and 0.05% (w/v) Tween 20 overnight at room temperature. Membranes were then incubated overnight at 4°C with rabbit polyclonal anti-GARNL1 (TULIP1) in a 1∶1000 dilution (Santa Cruz Biotechnology, CA, USA). Membranes were washed three times in TBST containing 0.05% Tween 20 and incubated in HRP-conjugated secondary antibody for 1 h at 37°C. Membranes were washed as before and signals were detected using Super ECL Detection Reagent (Applygen, Beijing, China) and Kodak (Kodak Film, USA).

Using cDNA transcribed from RNA pool composed of 6 chicken tissues as a template, the *GARNL1* ORF was amplified by primer pairs (P#4, Table S5) on the basis of the predicted mRNA sequence (accession number: XM421244). PCR was performed in a 50 µL reaction mixture containing 1 µL of KOD FX polymerase (Toyobo, Osaka, Japan), 25 µL of the 2×PCR buffer supplied by the manufacturer, and 10 µL of 2 mM dNTPs. The PCR conditions were 4 min at 94°C, followed by 30 cycles of 10 s at 98°C, 7 min at 68°C, and a final extension of 10 min at 68°C in a Bio-Rad S1000 (Bio-Rad Laboratories, Hercules, CA, USA). The amplified fragments were cloned into a pGEM-T Easy plasmid vector (Promega, Madison, USA) and then sequenced by Invitrogen Co. Ltd (Shanghai, China) via a commercial service. The obtained sequences were assembled to obtain the full-length *GARNL1* cDNA sequence. After sequencing, some novel alternative splice forms of the chicken *GANRL1* gene were detected by Reverse Transcription-PCR.

Sequence databases were accessed and searched using the BLAST algorithm at the NCBI at http://www.ncbi.nlm.nih.gov/BLAST and the UCSC Chicken Genome Project Working Draft at http://genome.ucsc.edu/. Protein domain predictions were obtained using the SMART program (Simple Modular Architecture Research Tool) from the European Molecular Biology Laboratory at http://smart.emblheidelberg.de/, and transmembrane helics motif and leucine zipper domain predictions were obtained using the SOSUI algorithm at http://bp.nuap.nagoya-u.ac.jp/sosui/sosui_submit.html and the PSORT II program at http://psort.ims.u-tokyo.ac.jp/form2.html. Multiple alignments of amino acid sequences were performed using ClustalW at http://www.ebi.ac.uk/clustalw/ and DNAMAN software (Lynnon corporation, Quebec, Canada), with MEGA 4.1 software (http://www.megasoftware.net/) used for homology analysis.

## Supporting Information

Figure S1 qPCR results of ten putatively differentially expressed transcripts identified by SSH.
(TIF)

Figure S2 Genes distributed in the block composed of GgaluGA282818 and rs14533299.
(TIF)

Figure S3 Partial cDNA and deduced amino acid sequence of *GARNL1-v1*, *GARNL1-v3*, *GARNL1-v4*.
(TIF)

Figure S4 GARNL1 protein sequences alignment among three species.
(TIF)

Table S1 Different expression gene identified by SSH.
(XLS)

Table S2 GO distribution of different expression gene.
(XLS)

Table S3 SNPs associated with EN300 trait in two-tail GWAS.
(XLS)

Table S4 The multiple comparisons of chicken EN300 traits and AFE trait in rs14532787.
(XLS)

Table S5 Primers used in present study.
(XLS)

Table S6 384 SNPs used for Illunima array.
(XLS)

## References

[1] Liu G, Dunnington EA, Siegel PB (1995). Correlated responses to long-term divergent selection for eight-week body weight in chickens: growth, sexual maturity, and egg production.. Poult Sci.

[2] Yang N, Jiang RS (2005). Recent advances in breeding for quality chickens.. World's Poult Sci J.

[3] Sharp PJ (1993). Photoperiodic control of reproduction in the domestic hen.. Poult Sci.

[4] Kuo YM, Shiue YL, Chen CF, Tang PC, Lee YP (2005). Proteomic analysis of hypothalamic proteins of high and low egg production strains of chickens.. Theriogenology.

[5] Klenke U, Constantin S, Wray S (2010). Neuropeptide Y directly inhibits neuronal activity in a subpopulation of gonadotropin-releasing hormone-1 neurons via Y1 receptors.. Endocrinology.

[6] Onagbesan OM, Metayer S, Tona K, Williams J, Decuypere E (2006). Effects of genotype and feed allowance on plasma luteinizing hormones, follicle-stimulating hormones, progesterone, estradiol levels, follicle differentiation, and egg production rates of broiler breeder hens.. Poult Sci.

[7] Sharp PJ (2005). Photoperiodic regulation of seasonal breeding in birds.. Ann N Y Acad Sci.

[8] Bhatt R, Youngren O, Kang S, El Halawani ME (2003). Dopamine infusion into the third ventricle increases gene expression of hypothalamic vasoactive intestinal peptide and pituitary prolactin and luteinizing hormone beta subunit in the turkey.. Gen Comp Endocrinol.

[9] Chaiseha Y, Youngren O, Al-Zailaie K, El Halawani ME (2003). Expression of D1 and D2 dopamine receptors in the hypothalamus and pituitary during the turkey reproductive cycle: colocalization with vasoactive intestinal peptide.. Neuroendocrinology.

[10] Youngren OM, Chaiseha Y, El Halawani ME (1998). Regulation of prolactin secretion by dopamine and vasoactive intestinal peptide at the level of the pituitary in the turkey.. Neuroendocrinology.

[11] Sharp PJ, MacNamee MC, Talbot RT, Sterling RJ, Hall TR (1984). Aspects of the neuroendocrine control of ovulation and broodiness in the domestic hen.. J Exp Zool.

[12] El Halawani ME, Rozenboim I (1993). The ontogeny and control of incubation behavior in turkeys.. Poult Sci.

[13] Sharp PJ, Sterling RJ, Talbot RT, Huskisson NS (1989). The role of hypothalamic vasoactive intestinal polypeptide in the maintenance of prolactin secretion in incubating bantam hens: observations using passive immunization, radioimmunoassay and immunohistochemistry.. J Endocrinol.

[14] Buntin JD, Lea RW, Figge GR (1988). Reductions in plasma LH concentration and testicular weight in ring doves following intracranial injection of prolactin or growth hormone.. J Endocrinol.

[15] El Halawani ME, Burke WH, Dennison PT (1980). Effect of nest-deprivation on serum prolactin level in nesting female turkeys.. Biol Reprod.

[16] Romanov MN, Talbot RT, Wilson PW, Sharp PJ (2002). Genetic control of incubation behavior in the domestic hen.. Poult Sci.

[17] Richard-Yris MA, Guemene D, Lea RW, Sharp PJ, Bedecarrats G (1998). Behaviour and hormone concentrations in nest deprived and renesting hens.. Br Poult Sci.

[18] Dunn IC, Miao YW, Morris A, Romanov MN, Wilson PW (2004). A study of association between genetic markers in candidate genes and reproductive traits in one generation of a commercial broiler breeder hen population.. Heredity.

[19] Xu HP, Shen X, Zhou M, Luo CL, Kang L (2010). The dopamine D2 receptor gene polymorphisms associated with chicken broodiness.. Poult Sci.

[20] El-Halawani ME, Whiting SE, Silsby JL, Pitts GR, Chaiseha Y (2000). Active immunization with vasoactive intestinal peptide in turkey hens.. Poult Sci.

[21] Mauro LJ, Elde RP, Youngren OM, Phillips RE, El Halawani ME (1989). Alterations in hypothalamic vasoactive intestinal peptide-like immunoreactivity are associated with reproduction and prolactin release in the female turkey.. Endocrinology.

[22] Cui JX, Du HL, Liang Y, Deng XM, Li N (2006). Association of polymorphisms in the promoter region of chicken prolactin with egg production.. Poult Sci.

[23] Jiang RS, Xu GY, Zhang XQ, Yang N (2005). Association of polymorphisms for prolactin and prolactin receptor genes with broody traits in chickens.. Poult Sci.

[24] Liang Y, Cui J, Yang G, Leung FC, Zhang X (2006). Polymorphisms of 5′ flanking region of chicken prolactin gene.. Domest Anim Endocrinol.

[25] Zhou M, Du Y, Nie Q, Liang Y, Luo C (2010). Associations between polymorphisms in the chicken VIP gene, egg production and broody traits.. Br Poult Sci.

[26] Zhou M, Lei M, Rao Y, Nie Q, Zeng H (2008). Polymorphisms of vasoactive intestinal peptide receptor-1 gene and their genetic effects on broodiness in chickens.. Poult Sci.

[27] Xu H, Shen X, Zhou M, Fang M, Zeng H (2010). The genetic effects of the dopamine D1 receptor gene on chicken egg production and broodiness traits.. BMC Genet.

[28] Dunn IC, Sharp PJ (1999). Photo-induction of hypothalamic gonadotrophin releasing hormone-I mRNA in the domestic chicken: a role for oestrogen?. J Neuroendocrinol.

[29] Ciccone NA, Dunn IC, Boswell T, Tsutsui K, Ubuka T (2004). Gonadotrophin inhibitory hormone depresses gonadotrophin alpha and follicle-stimulating hormone beta subunit expression in the pituitary of the domestic chicken.. J Neuroendocrinol.

[30] Jiang RS, Xu GY, Li JY, Wang XL, Yang N (2007). Associations of 24-bp Indel in PRL 5′-region with serum PRL level and laying performance in non-broody chickens.. Journal of China Agricultural University.

[31] Dunn IC, Joseph NT, Bain M, Edmond A, Wilson PW (2009). Polymorphisms in eggshell organic matrix genes are associated with eggshell quality measurements in pedigree Rhode Island Red hens.. Anim Genet.

[32] Uemoto Y, Suzuki C, Sato S, Sato S, Ohtake T (2009). Polymorphism of the ovocalyxin-32 gene and its association with egg production traits in the chicken.. Poult Sci.

[33] Xu HP, Zeng H, Luo CL, Zhang DX, Wang Q (2011). Genetic effects of polymorphisms in candidate genes and the QTL region on chicken age at first egg.. BMC genetic.

[34] Schutz K, Kerje S, Carlborg O, Jacobsson L, Andersson L (2002). QTL analysis of a red junglefowl x White Leghorn intercross reveals trade-off in resource allocation between behavior and production traits.. Behav Genet.

[35] Tuiskula-Haavisto M, Honkatukia M, Vilkki J, de Koning DJ, Schulman NF (2002). Mapping of quantitative trait loci affecting quality and production traits in egg layers.. Poult Sci.

[36] Tuiskula-Haavisto M, de Koning DJ, Honkatukia M, Schulman NF, Maki-Tanila A (2004). Quantitative trait loci with parent-of-origin effects in chicken.. Genet Res.

[37] Wardecka B, Olszewski R, Jaszczak K, Zieba G, Pierzchala M (2002). Relationship between microsatellite marker alleles on chromosomes 1–5 originating from the Rhode Island Red and Green-legged Partrigenous breeds and egg production and quality traits in F(2) mapping population.. J Appl Genet.

[38] Sasaki O, Odawara S, Takahashi H, Nirasawa K, Oyamada Y (2004). Genetic mapping of quantitative trait loci affecting body weight, egg character and egg production in F2 intercross chickens.. Anim Genet.

[39] Hansen C, Yi N, Zhang YM, Xu S, Gavora J (2005). Identification of QTL for production traits in chickens.. Anim Biotechnol.

[40] Schreiweis MA, Hester PY, Settar P, Moody DE (2006). Identification of quantitative trait loci associated with egg quality, egg production, and body weight in an F2 resource population of chickens.. Anim Genet.

[41] Sharp PJ (2004). Genes for persistency of egg laying: White Leghorns and broodiness.. Roslin Institute Edinburgh Annual Report.

[42] Basheer A, Wilson PW, Talbot RJ, Sharp PJ, Law A (2010). Dissecting the genetics of maternal behavior in chickens..

[43] Abasht B, Lamont SJ (2007). Genome-wide association analysis reveals cryptic alleles as an important factor in heterosis for fatness in chicken F2 population.. Anim Genet.

[44] Bouatia-Naji N, Bonnefond A, Cavalcanti-Proenca C, Sparso T, Holmkvist J (2009). A variant near MTNR1B is associated with increased fasting plasma glucose levels and type 2 diabetes risk.. Nat Genet.

[45] Schulze TG, Detera-Wadleigh SD, Akula N, Gupta A, Kassem L (2009). Two variants in Ankyrin 3 (ANK3) are independent genetic risk factors for bipolar disorder.. Mol Psychiatry.

[46] Rioux JD, Xavier RJ, Taylor KD, Silverberg MS, Goyette P (2007). Genome-wide association study identifies new susceptibility loci for Crohn disease and implicates autophagy in disease pathogenesis.. Nat Genet.

[47] Hasenstein JR, Hassen AT, Dekkers JC, Lamont SJ (2008). High resolution, advanced intercross mapping of host resistance to Salmonella colonization.. Dev Biol (Basel).

[48] Jorgenson E, Witte JS (2006). A gene-centric approach to genome-wide association studies.. Nat Rev Genet.

[49] Hemminger BM, Saelim B, Sullivan PF (2006). TAMAL: an integrated approach to choosing SNPs for genetic studies of human complex traits.. Bioinformatics.

[50] Shiue YL, Chen LR, Chen CF, Chen YL, Ju JP (2006). Identification of transcripts related to high egg production in the chicken hypothalamus and pituitary gland.. Theriogenology.

[51] Chen LR, Chao CH, Chen CF, Lee YP, Chen YL (2007). Expression of 25 high egg production related transcripts that identified from hypothalamus and pituitary gland in red-feather Taiwan country chickens.. Anim Reprod Sci.

[52] Chen CF, Shiue YL, Yen CJ, Tang PC, Chang HC (2007). Laying traits and underlying transcripts, expressed in the hypothalamus and pituitary gland, that were associated with egg production variability in chickens.. Theriogenology.

[53] Ahn JS, Moon SH, Kim J, Chung HM, Kim JK (2010). Identification of differentially expressed genes in human embryonic stem cell-derived endothelial cells using suppression subtractive hybridization.. Stem Cells Dev.

[54] Bao B, Xu WH (2011). Identification of gene expression changes associated with the initiation of diapause in the brain of the cotton bollworm, Helicoverpa armigera.. BMC Genomics.

[55] Gorni C, Garino C, Iacuaniello S, Castiglioni B, Stella A (2010). Transcriptome analysis to identify differential gene expression affecting meat quality in heavy Italian pigs.. Anim Genet.

[56] Schwarzbraun T, Vincent JB, Schumacher A, Geschwind DH, Oliveira J (2004). Cloning, genomic structure, and expression profiles of TULIP1 (GARNL1), a brain-expressed candidate gene for 14q13-linked neurological phenotypes, and its murine homologue.. Genomics.

[57] Shimojima K, Komoike Y, Tohyama J, Takahashi S, Páez MT (2009). TULIP1 (RALGAPA1) haploinsufficiency with brain development delay.. Genomics.

[58] Garwood VA, Lowe PC (1975). Responses in egg weight and rate of lay to bidirectional selection for age at maturity.. Poultry Sci.

[59] Heng JI, Tan SS (2002). Cloning and characterization of GRIPE, a novel interacting partner of the transcription factor E12 in developing mouse forebrain.. J Biol Chem.

[60] Hattori M, Tsukamoto N, Nur-e-Kamal MS, Rubinfeld B, Iwai K (1995). Molecular cloning of a novel mitogen-inducible nuclear protein with a Ran GTPase-activating domain that affects cell cycle progression.. Mol Cell Biol.

[61] Zhang K, Papageorge AG, Martin P, Vass WC, Olah Z (1991). Heterogeneous amino acids in Ras and Rap1A specifying sensitivity to GAP proteins.. Science.

[62] Mochizuki N, Ohba Y, Kiyokawa E, Kurata T, Murakami T (1999). Activation of the ERK/MAPK pathway by an isoform of rap1GAP associated with G alpha(i).. Nature.

[63] Elkin RG, Zhong Y, Porter RJ, Walzem RL (2003). Validation of a modified PCR-based method for identifying mutant restricted ovulator chickens: substantiation of genotypic classification by phenotypic traits.. Poult Sci.

[64] Han Buhm, Kang Hyun Min, Eskin Eleazar (2009). Rapid and accurate multiple testing correction and power estimation for millions of correlated markers.. PLoS Genet.

[65] Barrett JC, Fry B, Maller J, Daly MJ (2005). Haploview: analysis and visualization of LD and haplotype maps.. Bioinformatics.

