# Supplementary figures and images for: The GTPase Activating Rap/RanGAP Domain-Like 1 Gene Is Associated with Chicken Reproductive Traits
Source: PLoS One. 2012 Apr 9;7(4):e33851. doi: 10.1371/journal.pone.0033851 (PMC3322132; doi:10.1371/journal.pone.0033851)

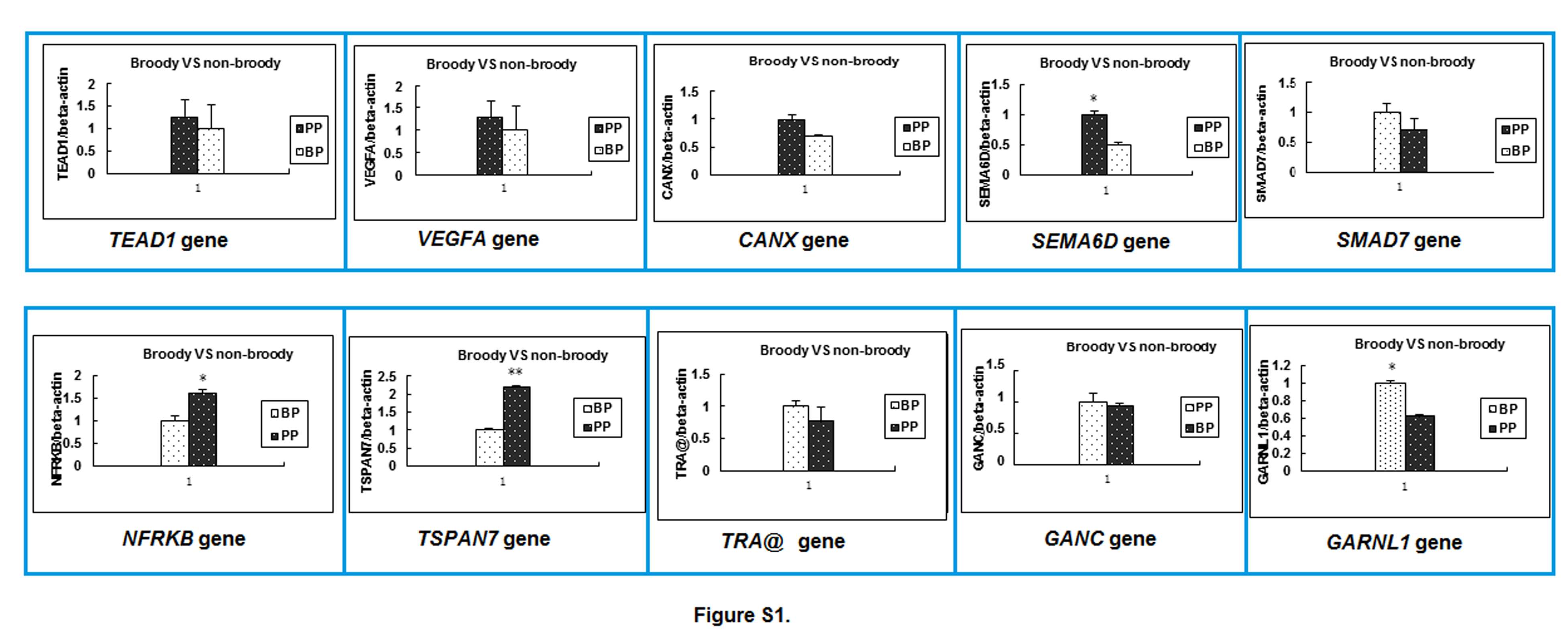

Supplement: Figure S1 — qPCR results of ten putatively differentially expressed transcripts identified by SSH. (TIF) [file pone.0033851.s001.tif]

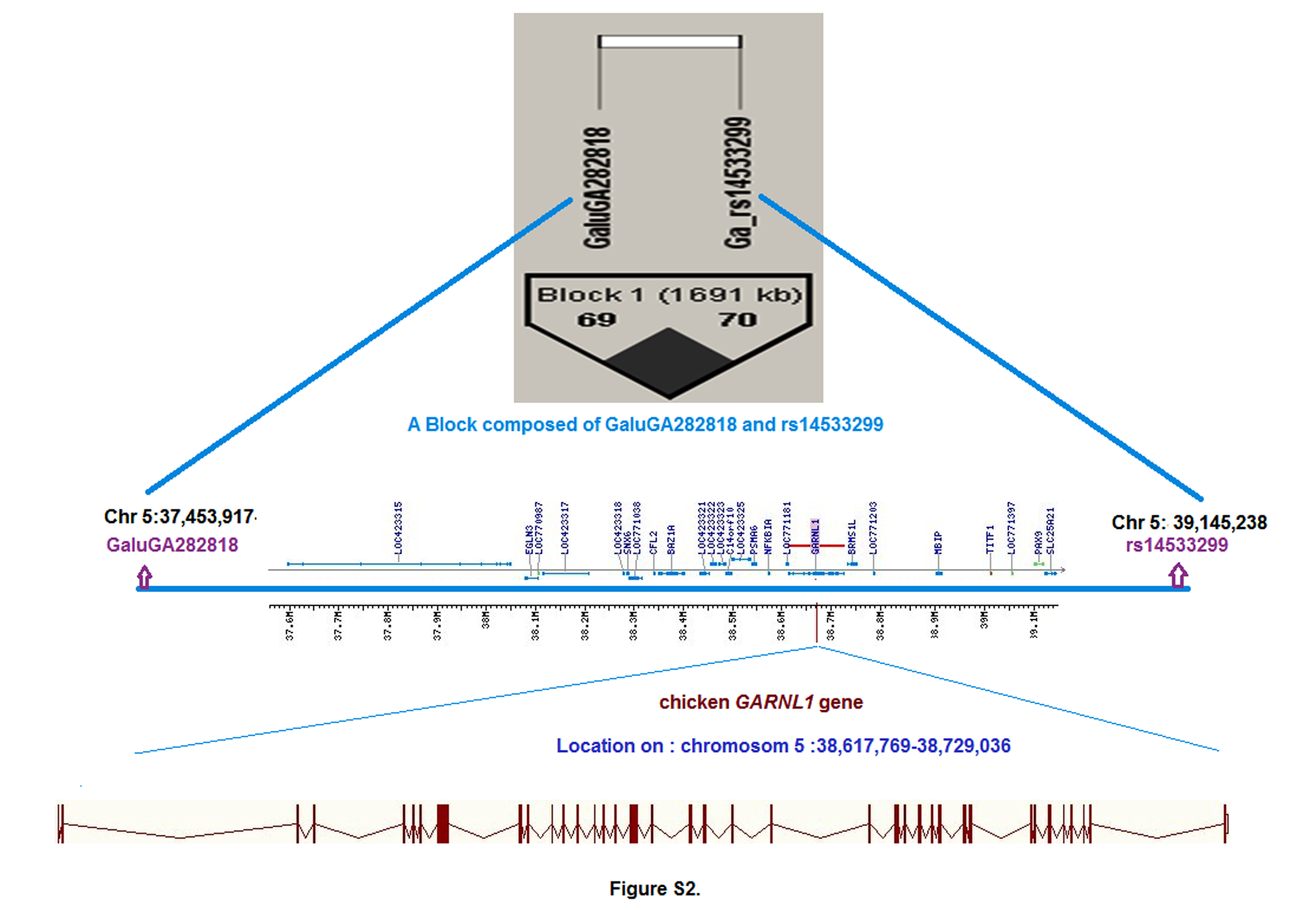

Supplement: Figure S2 — Genes distributed in the block composed of GgaluGA282818 and rs14533299. (TIF) [file pone.0033851.s002.tif]

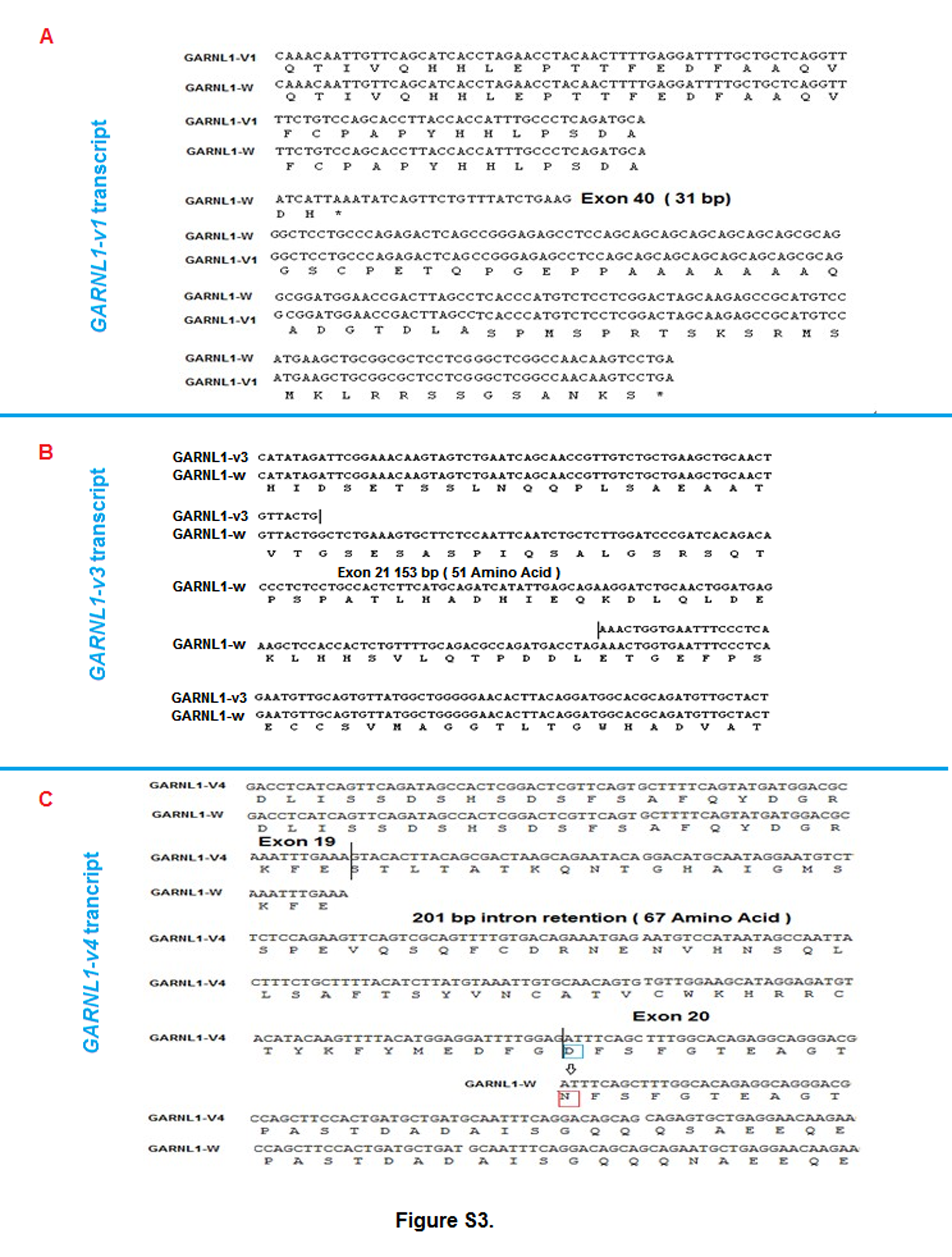

Supplement: Figure S3 — Partial cDNA and deduced amino acid sequence of GARNL1-v1, GARNL1-v3, GARNL1-v4. (TIF) [file pone.0033851.s003.tif]

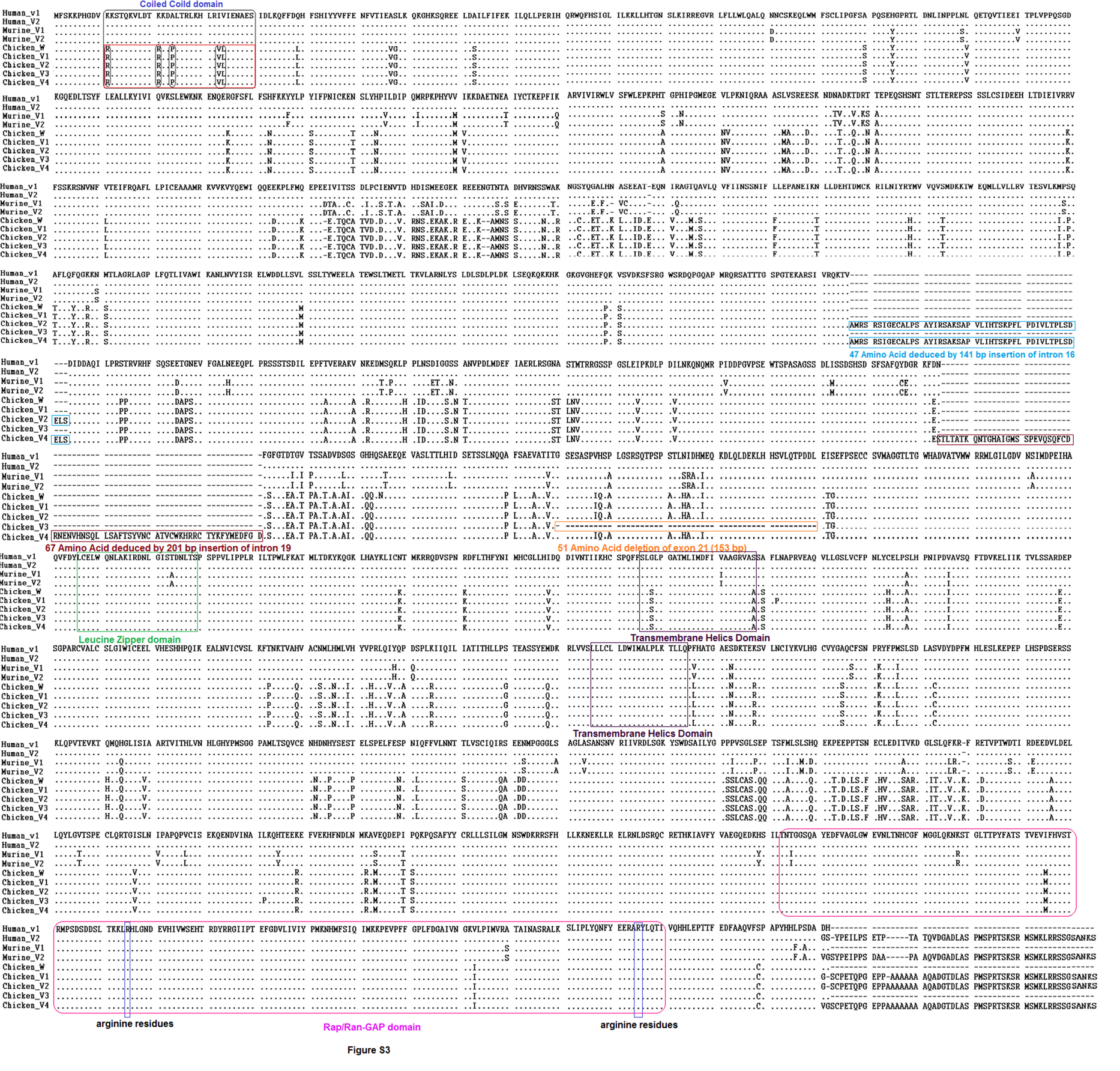

Supplement: Figure S4 — GARNL1 protein sequences alignment among three species. (TIF) [file pone.0033851.s004.tif]
